# Visceral Adipose Tissue: The Hidden Culprit for Type 2 Diabetes

**DOI:** 10.3390/nu16071015

**Published:** 2024-03-30

**Authors:** Sneha Dhokte, Krzysztof Czaja

**Affiliations:** Department of Biomedical Sciences, College of Veterinary Medicine, University of Georgia, Athens, GA 30602, USA; sneha.dhokte@uga.edu

**Keywords:** visceral adipose tissue, type 2 diabetes, obesity, free fatty acids, body mass index, body composition

## Abstract

Type 2 diabetes (T2D) is a chronic metabolic disorder characterized by insulin resistance in various tissues. Though conventionally associated with obesity, current research indicates that visceral adipose tissue (VAT) is the leading determining factor, wielding more influence regardless of individual body mass. The heightened metabolic activity of VAT encourages the circulation of free fatty acid (FFA) molecules, which induce insulin resistance in surrounding tissues. Individuals most vulnerable to this preferential fat deposition are older males with ancestral ties to Asian countries because genetics and sex hormones are pivotal factors for VAT accumulation. However, interventions in one’s diet and lifestyle have the potential to strategically discourage the growth of VAT. This illuminates the possibility that the expansion of VAT and, subsequently, the risk of T2D development are preventable. Therefore, by reducing the amount of VAT accumulated in an individual and preventing it from building up, one can effectively control and prevent the development of T2D.

## 1. Introduction

Diabetes mellitus is a chronic metabolic disease that has increased dramatically in recent years. As of 2020, the disease was found in 11.3% of Americans (referring to individuals in the United States of America for the purpose of this text), with 90–95% of cases being categorized as insulin-resistant (Type 2) [1]. This alarming prevalence rate creates growing concern when considering that it has more than doubled in just a few decades, as, in the mid-1970s, only 5.3% of Americans had been recorded as diabetic [2]. Type 2 diabetes (T2D) is commonly attributed to obesity, though it poses a significant threat to non-obese individuals, constituting 10–20% of T2D cases worldwide [1]. Additionally, roughly 41.9% of Americans are considered obese, while 30% of Americans are categorized as obese but do not express T2D [1,3]. This shows that approximately 12% of Americans are obese and also suffer from T2D. While a connection between the two is present, the data further supports the idea that obesity is not the strongest indicator of T2D. Fat distribution, specifically VAT distribution, has emerged as a key determinant for whether or not an individual will develop T2D due to the tissue’s insulin resistance. This review aims to explore the current literature regarding the impact of VAT on the development of T2D. It will assess VAT in comparison to other forms of adipose tissue, investigate its role in encouraging insulin resistance, uncover various factors that promote its formation, and evaluate optimal measurement methods. Gaining a deeper understanding of the negative effects of VAT is imperative for suppressing the global surge of T2D cases and preserving metabolic health.

Diabetes is characterized by hyperglycemia, which results from issues with insulin production and/or sensitivity [4]. This condition is typically diagnosed by a fasting plasma glucose test that shows a result of ≥126 mg/dL or a 2 h plasma glucose test that shows a result of ≥200 mg/dL [5]. Some labs may also utilize a heavily standardized A1C test, which indicates diabetes in patients who have results ≥6.5% [5]. These abnormally high blood sugar values can give rise to complications that are categorized as microvascular or macrovascular. Microvascular problems involve damage to small vessels in organ systems such as the nervous and renal systems, as well as individual organs such as the eye [6]. In contrast, macrovascular issues are characterized by damage to large vessels, which can result in heart attacks, strokes, congestive heart failure, and several others [6]. Thankfully, there are numerous preventable risk factors on which individuals can focus to minimize the risk that they will develop this condition. These include poor diet, sedentary lifestyles, smoking, alcohol consumption, and, most significantly, body fat composition [6]. There are two main types of diabetes, both of which have very different risk factors, prevalence rates, and complications.

Type 1 diabetes is a category of diabetes that is associated with insulin deficiency caused by the immune destruction of pancreatic β-cells [7,8]. The condition has three well-defined stages; stage 1 is characterized by autoimmunity, a normal glucose range, and a lack of symptoms [5]. Stage 2 continues to display autoimmunity and a lack of symptoms but also includes dysglycemia [5]. Finally, at stage 3, patients are symptomatic and new-onset hyperglycemia is present [5]. After diagnosis, about 90% of patients have significant antibodies against β-cell proteins, which include insulin [8]. This autoimmune response to insulin forces excessive amounts of glucose to build up in a patient’s blood.

Type 2 diabetes makes up the overwhelming majority of diabetes cases and is characterized by insulin resistance in several tissues, including muscle, liver, and adipose tissue [4,5,7,9]. Insulin sensitivity decreases due to continual secretion, which leads to hyperinsulinemia in an effort to compensate for and prevent hyperglycemia [9]. However, this cannot be sustained, and β-cell function declines over time, resulting in defective insulin secretion and subsequently increased blood sugar levels [9]. This increase can lead to a multitude of complications that involve various organs/organ systems (Table 1). Patients may experience improvement with medication and weight loss, but insulin resistance typically cannot be reversed [5]. One of the biggest risk factors for T2D is obesity, which makes understanding the relationship between the two even more important [9]. 

Obese individuals are more likely to develop T2D than their healthy-weighted counterparts for a multitude of reasons. Obese individuals secrete more insulin after consuming glucose, which leads to hyperinsulinemia [23,24]. Because of this, they are more likely to experience complications with pancreatic β-cell function and insulin resistance [23,24]. Individuals with obesity also have significant amounts of adipose tissue. Adipose tissue corresponds with an increased presence of certain fatty acids, glycerol, hormones, and cytokines that promote inflammation; all of these have been shown to alter insulin sensitivity and lead to resistance [24,25]. So, while obesity poorly predicts an eventual diagnosis of T2D, it does increase the risk of developing this condition. There is also evidence of the opposite, where T2D can contribute to obesity. Researchers found that individuals who are genetically predisposed to T2D may become obese over time due to their natural susceptibility to insulin resistance [26]. This is because they have abnormal amounts of glucose and dysfunctional insulin in their bloodstream, which results in adipose tissue accumulation [26]. In order to stop these individuals from joining the 41.9% of Americans who are characterized as obese, it is necessary to understand the link between obesity and preventative measures such as controlling dietary intake [1]. 

Energy balance refers to the equilibrium between energy intake and expenditure; it may also be referred to as “calories in equals calories out” when trying to maintain weight [27,28]. Because this balance is so delicate, even a small variance in caloric intake or output can result in significant changes to an individual’s body weight [27]. Calorie overconsumption is described as when an individual’s calorie intake exceeds their energy expenditure [29]. Though short-term overconsumption is not generally very harmful, when overconsumption is carried on for extended periods of time, the body begins to gain weight [27,29]. This is because once the body has consumed the calories necessary for energy balance, the excess calories are stored in fat cells within the body [27]. These fat cells grow and multiply to form large amounts of adipose tissue, which stores energy for later use [30]. Over time, this adipose tissue will accumulate, and because of this, individuals who consume excessive amounts of calories will progressively gain weight; without any dietary intervention, they will likely become overweight or clinically obese.

Calorie intake and T2D are closely intertwined because increased calorie intake leads to obesity, which puts individuals at a much greater risk for T2D. Essentially, calorie overconsumption leads to adipose tissue growth, which results in not only weight gain but also insulin resistance; both of these are driving forces behind T2D [24]. A series of surveys revealed that the average American currently consumes about 3864 kcal/day, which is greater than any other country’s citizens [31]. This is a substantial increase from previous decades, such as the late 1970s, when the average adult male consumed 1866–2568 kcal/day and the average adult female consumed 1417–1748 kcal/day [32]. Many people have attributed this increase in caloric intake to being one of the driving factors behind the rise of T2D cases. Using this connection, researchers have discovered that calorie-deficit diets may be able to control the disease and, in some cases, even put it into remission [33]. The two types of calorie deficit diets assessed were low-calorie diets (LCDs) and very low-calorie diets (VLCDs); LCDs are usually used as a relative term, while VLCDs are consistently between 400 and 800 kcal/day [33]. Though they may seem drastic, one study found that about 40% of T2D patients went into remission after six months of following a LCD, and another showed that 79% of remissions were achieved after 8–12 weeks on a VLCD [33]. This is because LCDs have been proven to lower fasting plasma glucose levels, and VLCDs lower glycated hemoglobin (A1C) levels for the long-term future [33,34]. Calorie intake has a significant effect on T2D, so gaining a deeper understanding of this relationship is likely to allow researchers to discover even more treatment and prevention options.

Over the past few decades, body mass index (BMI) has become the most widely used measurement for quickly assessing an individual’s metabolic health because it can easily classify individuals as underweight, normal, overweight, or obese. This index has frequently been utilized to assess a patient’s risk for chronic conditions that include T2D and hypertension [35]. While a simple and straightforward calculation like BMI can be extremely valuable, especially for individuals lacking access to healthcare, it has several limitations. The most significant of these is that it is unable to account for variables other than height and weight, such as muscle or fat composition [35]. Because of this, an individual with significant muscle mass may be categorized as obese, while another with significant body fat may fall into the healthy category. Taking these factors into account is imperative when analyzing chronic conditions such as T2D since, while obesity is commonly associated with the onset of T2D, it is not necessary for an individual to develop it. Approximately 10–20% of diabetes cases correspond to non-obese individuals (assessed by BMI) around the world, and this proportion markedly increases to 60–80% in several Asian countries [3]. This is especially common in countries such as India and mainland China, which have the highest number of non-obese T2D patients [3]. As a result, hundreds of millions of people are at risk for various metabolic illnesses despite externally appearing to be in good condition. Additionally, despite an overwhelming recent increase in obesity and the number of obese T2D patients, as of 2019, 57.9% of T2D-related deaths worldwide were attributed to patients whose BMI categorized them as non-obese [19]. Non-obese T2D has also proven to have significantly higher age-standardized mortality rates in certain regions, such as Oceania, Central sub-Saharan Africa, Eastern sub-Saharan Africa, Southeast Asia, Western sub-Saharan Africa, and South Asia, compared with obese T2D [19]. This information points to the conclusion that BMI is not the most important indicator of T2D development, with possible other factors playing more significant roles. These statistics indicate that there are numerous other factors that can play a role in T2D onset other than patient weight, and this “outlier” group of T2D cases may be even more dangerous. One of the factors that has been commonly identified has been the presence of increased VAT, which is a type of adipose tissue that accumulates in the mesentery and omentum of the abdomen [36].

The argument for body fat composition being superior to BMI in determining T2D susceptibility is supported by the fact that BMI-obesity does not guarantee a T2D diagnosis. As already indicated, 41.9% of Americans are obese, but the T2D prevalence rate is only 11.3% [1]. This leaves roughly 30% of Americans who are obese and will never have to struggle with the condition despite their weight. It is important to note that this proportion may be lower when taking into consideration the number of individuals who have T2D but are yet to be diagnosed. Many of these individuals fall under a category referred to as “metabolically healthy obesity” (MHO). MHO is loosely used to describe a group of people who are considered obese by BMI but do not exhibit any metabolic or cardiovascular issues, such as T2D [37]. Despite their BMI, people with MHO do not often gain many health benefits from losing weight, though they are advised not to gain additional weight [37]. This is further evidence that other characteristics may be more significant for predicting the risk of T2D than BMI. Though MHO is associated with several phenotypic characteristics, one of the most relevant is that individuals with MHO have less VAT, less liver fat, and more lower body subcutaneous fat compared with their counterparts with metabolically unhealthy obesity (MUO) [38]. This once again indicates that adipose tissue deposition is one of the most significant factors when it comes to the development of T2D. Due to this, assessing an individual’s body composition would be more useful than their BMI as a tool for preventing the rise in T2D cases. This review will provide insight into how body composition, specifically preferential VAT deposition, affects the development of T2D. It will additionally address how various factors influence the accumulation of VAT as opposed to other adipose tissue types. 

## 2. Why Only Visceral Adipose Tissue?

Adipose tissue is a complex, metabolically active organ made of many cells serving various functions. Differences in types of adipose tissue and the location of their deposit can vastly change its impact on the body. Visceral adipose tissue is a type of white adipose tissue characterized by large adipocytes and is linked to poor metabolic health and worse health outcomes overall [39]. It is found in the abdominal cavity near organs such as the intestines, liver, and pancreas, which makes it more involved in metabolic processes. This is contrasted by subcutaneous adipose tissue (“subcutaneous fat”), the other type of white adipose tissue, which is viewed as much more metabolically healthy. This adipose tissue is characterized by smaller adipocytes found directly below the skin that store energy and insulate the body [39]. Brown adipose tissue (“brown fat”) is the final type of adipose tissue and is seen as the most metabolically healthy overall due to its active role in thermogenesis [39]. It is termed “brown” due to the high abundance of mitochondria, which give it a darker appearance [39]. This type of adipose tissue is often found in the smallest quantities in select regions scattered around the human body but is still associated with more favorable health outcomes [39]. 

**Figure 1:**
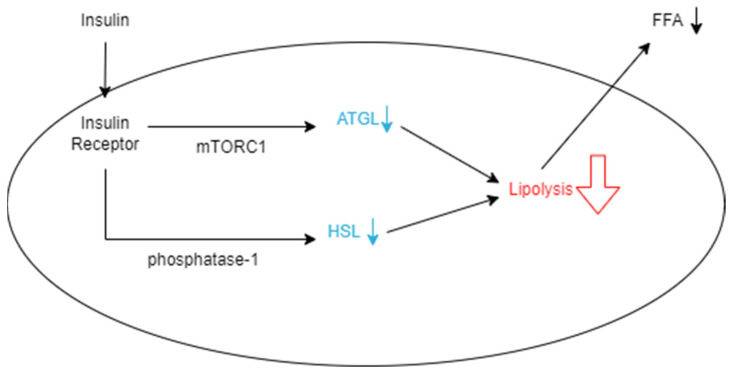
Schematic representation of how insulin decreases the rate of lipolysis in an adipocyte. Insulin binds to insulin receptors, which allows it to suppress ATGL expression through the mTORC1-mediated pathway. It also decreases HSL by dephosphorylation with phosphatase-1. When these enzymes are hindered, the rate of lipolysis in the adipocyte decreases, and fewer FFAs are released from the cell into the bloodstream.

A metabolic reason VAT may have high utility for predicting T2D is its decreased uptake and increased release of free fatty acids (FFAs), which are molecules known to cause insulin resistance when circulating in high concentrations. Subcutaneous fat contributes to adiposity, but it is generally deemed less influential on insulin resistance compared with VAT [40,41,42]. Subcutaneous adipocytes are small and insulin-sensitive compared with those in VAT. This leads to the cells acting as “sinks” or “buffers,” which can absorb FFAs and triglycerides that are circulating after calories have been consumed [36]. Visceral adipocytes, on the other hand, are much larger, more insulin-resistant, and more metabolically active [36]. Because they are more active, they have the ability to rapidly store fatty acids as triglycerides and release them as FFAs after undergoing lipolysis, while subcutaneous adipocytes hold on to triglycerides for longer periods of time [43]. However, despite releasing more FFAs, visceral adipocytes have also been shown to have lower rates of total tissue FFA uptake compared with subcutaneous adipocytes, which supports that they are accountable for increasing the concentration of FFAs [44]. As expanded on later, FFAs are heavily associated with impacting insulin sensitivity, so greater amounts of VAT are responsible for this occurrence. Visceral adipocytes are also described as insulin-resistant, which means that insulin does not have a significant effect on the cells’ processes. Insulin is capable of reducing the rate of lipolysis by suppressing the rate-limiting enzyme adipose triglyceride lipase (ATGL), which is responsible for many triglyceride, glycerol, and fatty acid activities (Figure 1) [45,46]. This may be done by posttranslational regulation of the enzyme through feeding, fasting, exercise, or other physiological aspects or regulation of ATGL expression through the mammalian target of rapamycin complex 1 (mTORC1)-mediated pathway, which decreases the transcription of ATGL [46]. However, when insulin is present in insulin-resistant tissues, FFAs continue to be released rapidly from VAT as lipolysis is unaffected [46]. The other significant enzyme responsible for lipolysis is hormone-sensitive lipase (HSL), which largely impacts the hydrolytic activity throughout the process [45]. When the enzyme is phosphorylated, it increases hydrolytic activity and thus increases the rate of lipolysis in adipocytes. Insulin inhibits this process by activating protein phosphatase-1, which acts against HSL by dephosphorylating it (Figure 1) [45]. Another consequence of the tissue’s insulin resistance is that insulin is unable to encourage glucose from entering the adipocyte and becoming oxidized [45]. As a result, instead of being converted into usable ATP, glucose molecules continue to circulate and increase an individual’s blood sugar [36]. Essentially, insulin works to decrease the rate of lipolysis so that fewer FFAs are released into the bloodstream to be taken up by VAT. This ensures that VAT is minimized and insulin resistance is prevented in various tissues that take up FFAs. 

Visceral and subcutaneous fat are both categorized as white adipose tissue, which is a common group of tissues that contribute to adiposity. This differs in several regards from brown adipose tissue, a type of tissue that may be beneficial for some individuals due to its metabolic activity. Brown adipose tissue is extremely significant when it comes to thermogenesis and energy expenditure because it contains a greater abundance of mitochondria [47]. As a result, it is also better equipped to metabolize glucose, uptake FFAs, and generate heat, all while remaining relatively insulin-sensitive [47]. As white and brown adipose tissues undergo lipolysis, FFAs are released into the bloodstream and become available to various other tissues [48]. This may include brown adipose tissue, especially during periods of cold exposure, as these circulating lipids are heavily relied upon to generate heat [48]. When brown fat detects FFAs, it acts quickly to take up the molecules using fatty acid transport proteins, FFA3 proteins, and a cluster of differentiation scavenger receptor 36 (CD36) [49]. Once taken up, these fatty acids will either be stored as lipid droplets or transported to the mitochondrial matrix or peroxisome to undergo fatty acid oxidation and form acetyl-CoA [49]. This molecule helps yield NADH and FADH molecules through the tricarboxylic acid cycle, which promote proton transport to the mitochondrial intermembrane space to produce energy [49]. Uncoupling protein-1 (UCP1) works to dispel this proton gradient, release energy as heat, and contribute to thermogenesis [49]. Due to brown fat’s dependence on FFAs, it takes up many of the molecules for storage and fuel and reduces the number of circulating FFAs in the bloodstream. Since increased FFAs are associated with insulin resistance, the presence of certain adipose tissues, such as brown adipose tissue, may actually be beneficial despite contributing to adiposity and overall excess body weight. Though subcutaneous, visceral, and brown fat all contribute to adiposity, VAT has a strong correlation with insulin resistance due to its increased rate of lipolysis, lower rate of FFA uptake, and poor ability to store FFAs for extended periods of time. Subcutaneous and brown fat are much more beneficial, as they are effective at taking up circulating FFAs, and brown fat is more capable of utilizing them for thermogenesis. The type of adipose tissue accumulated in an individual’s body is significant as their characteristics vary greatly, with VAT being more closely linked to insulin sensitivity compared with subcutaneous or brown fat.

## 3. Visceral Adipose Tissue: The Cause of Insulin Resistance

**Figure 2:**
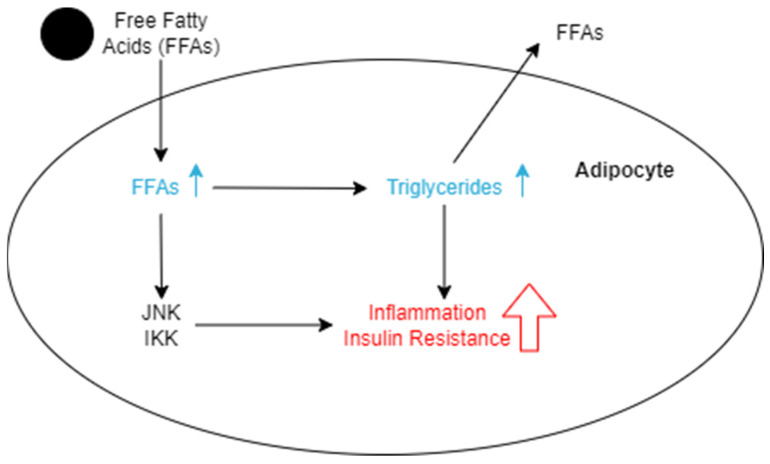
Schematic representation of the effects of FFAs entering an adipocyte. The increase of FFAs leads to the accumulation of triglycerides, which causes the cell to become inflamed and more insulin resistant. As the cells become too inflamed to hold the fat, lipolysis occurs, which breaks down the triglycerides into fatty acids, which are released back into the bloodstream. Increased levels of FFAs also increase the expression of the JNK and IKK pathways, which directly results in more inflammation and insulin resistance.

Since it has been established that VAT is most closely linked to insulin resistance out of all the adipose tissues, it is critical to understand the exact mechanisms that enable this and how it compares to other measures that predict T2D. Researchers in South Korea conducted a cross-sectional study assessing six different measures of obesity, including BMI, waist circumference, waist/height ratio, waist/hip ratio, waist/thigh ratio, and VAT. In doing this, they found that the amount of VAT in an individual was a significantly better indicator of diabetes than any of the others analyzed [41]. Their findings were backed by a study in Japan seeking to compare abdominal adiposity and cardiorespiratory fitness as factors in T2D development. This study also concluded that VAT was one of the most significant predictors of insulin resistance [40]. A conclusion of researchers from this is that VAT affects insulin metabolism throughout the body by releasing FFAs, as mentioned above, and decreases the rate of re-esterification [41,50]. Adipocytes are capable of expanding to store large amounts of fat and remaining healthy, but when their expansion is excessive, inflammation is induced (Figure 2). This inflammation occurs as a result of c-Jun N-terminal kinase (JNK) and IκB kinase (IKK) expression in the fat cell, which increases the development of insulin resistance in the adipocytes [51]. The insulin resistance will encourage lipolysis within the cell, and triglycerides will be broken down, releasing FFAs back into the bloodstream (Figure 2). This continues to induce insulin resistance in other tissues as FFAs are released into plasma from adipose tissue and travel to other sites such as the liver through the portal vein and skeletal muscle vasculature [51]. 

**Figure 3:**
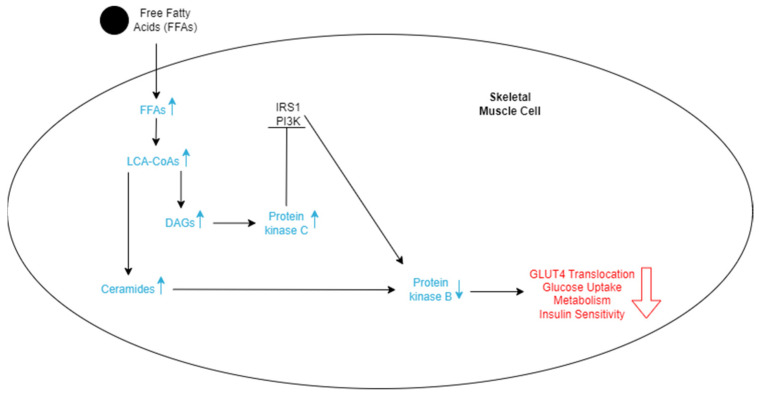
Schematic representation of the effects of FFAs entering skeletal cells. The increase in FFAs stimulates an increase in LCA-CoAs, which in turn increases the level of DAGs and ceramides. More ceramides lead to a decrease in protein kinase B. DAGs lead to an increase in protein kinase C, which inhibits the IRS1 and PI3K pathways, ultimately also decreasing protein kinase B. The lowered levels of protein kinase B decrease GLUT4 translocation, glucose uptake, metabolism of the cell, and insulin sensitivity, which promotes insulin resistance.

Metabolically healthy skeletal muscles store both glucose and FFAs as a source of energy. However, when there is an excess of FFAs in the bloodstream, skeletal muscle will uptake more of them, which may lead to an accumulation within muscle fibers [52]. When broken down, these stored fats are converted to metabolites such as long-chain acyl-CoAs (LCACoAs), ceramides, and diacylglycerols (DAGs), which are directly responsible for insulin resistance in these tissues (Figure 3) [52]. LCACoAs have been found to inhibit hexokinases, which are the first enzymes utilized in glucose metabolism [53]. They may also activate isoforms of protein kinase C, both directly and indirectly, by creating DAGs, which impair the function of the IRS-1 pathway by serine phosphorylation (Figure 3) [53]. This subsequently affects the PI3K pathway function and reduces skeletal muscle glucose intake and metabolism [53]. Ceramides decrease tyrosine phosphorylation of the IRS-1 pathway and inhibit phosphorylation of protein kinase B [53]. Both of these actions directly result in the reduction of GLUT4 translocation and, ultimately, less glucose uptake into skeletal muscles (Figure 3). 

Hepatic insulin resistance as a result of FFAs can be explained by three main things. One is that the mechanisms in place are very similar to those in skeletal muscles, where the insulin signal transduction system is inhibited by metabolites of FFAs [54]. The second is that since the liver is responsible for roughly 80% of endogenous glucose production, FFA oxidation acts as an energy source for gluconeogenesis [54]. This results in more glucose being released into the bloodstream and creates a positive feedback loop for insulin resistance. The third is similar to the second, as FFAs are taken up by liver cells and the rate of lipid oxidation increases [54]. This results in acetyl CoA buildup, and rate-limiting enzymes for gluconeogenesis and glucose release are stimulated [54]. The presence of VAT is now appearing to have a significant impact on various insulin-sensitive tissues because of its relationship with FFAs. FFAs are capable of inducing insulin resistance in various tissues, especially adipose tissue, skeletal muscle, and the liver, through many complex biochemical pathways. Because VAT directly impacts FFA release, it is important to understand how VAT forms and why some individuals are more at risk of accumulating it than others.

## 4. Unveiling Influences on Visceral Adipose Tissue

VAT is formed when an excess number of calories is consumed and broken down into macronutrients. Dietary fats, in particular, are broken down into smaller components, like FFAs, and released into the bloodstream [55,56]. These FFAs are either taken up and stored as triglycerides in subcutaneous fat deposits, or they will accumulate around organs and be taken up into VAT [55,56]. There are several factors that influence whether an individual will have more subcutaneous adipose tissue, or VAT.

### 4.1. Genetic Blueprint

Genetics, specifically relating to ethnicity and ancestry, plays a large part in fat deposits throughout the body. One study showed that individuals from China, Taiwan, and Hong Kong consistently had more subcutaneous and VAT, as did many participants from Bangladesh, India, Nepal, Pakistan, and Sri Lanka [57]. These individuals typically had more body fat at a given BMI compared with their counterparts from continental Europe, Ireland, and the United Kingdom, as well as those who describe themselves as Aboriginal [57]. Another study assessed individuals of different backgrounds in the United States and found that Japanese-Americans had the most mean VAT area, while African-Americans had the least [58]. This is significant because Japanese-Americans have a relatively low obesity prevalence rate of 8.7% compared with the obesity prevalence rate of African Americans, which is drastically higher at 49.5% [59]. These statistics reiterate how independent BMI and body fat composition are from each other, as a group that is generally non-obese is also able to have the greatest mean amounts of VAT. 

**Figure 4:**
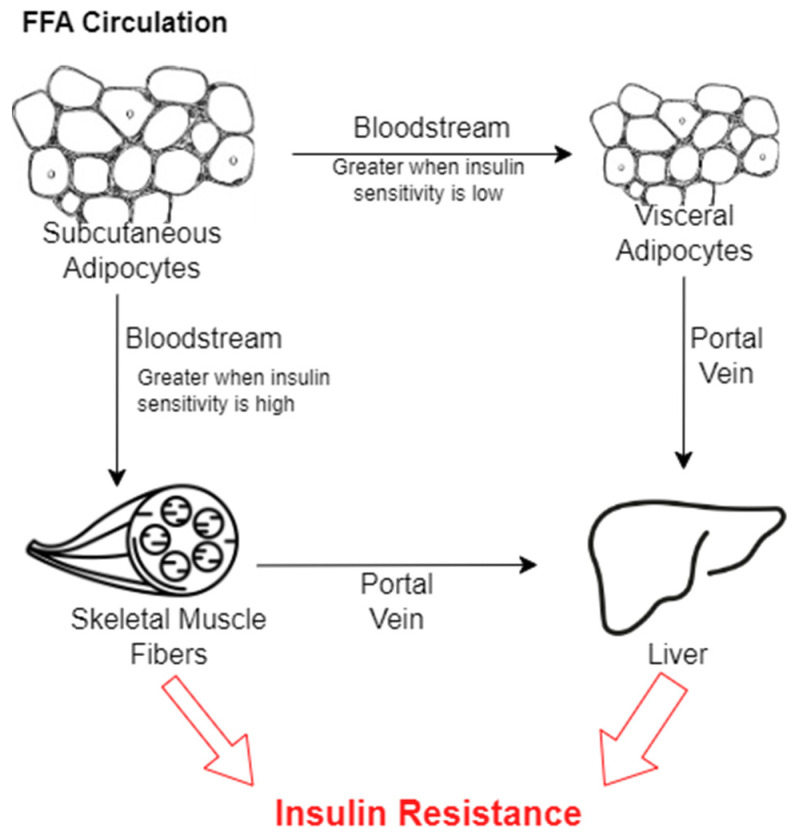
Schematic representation of the effects of FFAs circulating through the body. FFAs are initially taken up by subcutaneous adipocytes and then released into the bloodstream. Some will be taken up by skeletal muscle fibers, especially when insulin sensitivity is high, and others will be passed to visceral adipocytes, especially when insulin sensitivity is lower. FFAs released by visceral adipocytes will travel through the portal vein and enter the liver. FFAs released by skeletal muscle fibers will travel to and be taken up by the liver. This circulation of FFAs will eventually lead to insulin resistance in one or more of the tissues.

The trend of many individuals from countries in Asia having a high body fat content for their BMI is known as the “thin-fat phenomenon,” and it has been observed in numerous studies in regard to insulin resistance [60]. Because of how many diverse ethnic groups reside in these countries, it is difficult to identify one shared gene that could be responsible for this occurrence. Despite this, researchers strongly support the idea that genetics are largely responsible, as 16–25% of non-obese individuals with T2D have a family member who also has this metabolic disorder [3]. Though there is much uncertainty surrounding this phenomenon, one of the leading theories for why it occurs is referred to as the “adipose tissue expandability hypothesis”. It states that tissue such as subcutaneous adipose tissue will uptake lipids and expand until reaching a physiologic capacity, after which lipids will begin to accumulate in other regions such as VAT, skeletal muscle, or the liver (Figure 4) [61,62]. This has been more deeply explored in reference to sex differences, but it is also applicable to racial differences, though there is extensive potential to research it further. 

One study investigated the differences in FFA release from adipose tissue as it relates to various factors, including racial differences between African American and Caucasian participants [63]. This has been more deeply explored in reference to sex differences, but it is also applicable to racial differences, though there is extensive potential to research it further. The study ultimately found that palmitate (FFA) flux was similar between both groups, but plasma triglyceride concentrations were significantly lower in African American individuals compared with Caucasian individuals [63]. Triglycerides are complex molecules made up of FFAs that can be taken up by adipose tissues, so a decreased abundance of them in the bloodstream implies that a greater concentration has been taken up by peripheral tissues. It is important to reiterate that this study did not focus directly on the storage capacity of FFAs as strongly as other studies involving sex, but it is still significant because it implies that the role of race is valuable in preferential fat deposition. This study also only assessed variability between Caucasian and African American individuals, which leaves room for speculation on how members of other racial/ethnic groups store FFAs. For example, it is possible that people from Asian countries are at the lower end of the threshold for FFA storage in their subcutaneous adipose tissue, and, as a result, FFAs continue to travel through their bloodstream towards VAT deposits instead of being taken up by these tissues (Figure 4). This could provide a stronger basis for why the thin-fat phenomenon occurs. This is an understudied area for which extensive experimentation must be conducted before definitively concluding if hypotheses such as this may be correct. Despite the lack of understanding about exactly how it plays a role, genetics and ethnic background are often regarded as the most prominent factors in deciding where fat accumulates due to observed trends such as the “thin-fat phenomenon” and the potential for the adipose tissue expandability hypothesis. However, there are still numerous other aspects to consider when determining why fat deposits differently for different people.

### 4.2. Sexual Dichotomy

Sex and hormones are also extremely significant to VAT growth, but the underlying mechanisms for their influence remain in part unclear. Females tend to have more estrogen, which maintains their distribution of subcutaneous fat with limited abdominal VAT, leading to a “pear” shape. It does this by increasing the number of antilipolytic α2A-adrenergic receptors, which limits the lipolytic response to subcutaneous fat while VAT continues to be broken down [64]. Estrogen also works to enhance adipogenesis and encourage the differentiation of preadipocytes into mature subcutaneous adipocytes [65]. For this to occur, estrogen binds to estrogen receptors (ERα), which are found in greater abundance in subcutaneous adipose tissue [65]. Once bound, the receptor activates several signaling pathways to promote adipogenesis in the tissue, but since more receptors are found on subcutaneous fat, a greater number of preadipocytes are differentiated into subcutaneous adipose tissue [65]. This also prevents the growth of VAT, as described in the adipose tissue expandability hypothesis, because of the greater amounts of subcutaneous fat uptake of FFAs in the bloodstream instead of letting it continue toward VAT deposits (Figure 4). 

The effects of estrogen can be seen clearly in post-menopausal females, as they have much lower levels of estrogen, which decreases their capacity for FFA uptake in subcutaneous fat and thus increases VAT accumulation as they age [65]. Males, on the other hand, have many more androgens, such as testosterone, which promotes shrinking visceral adipocytes, which would overall reduce visceral adiposity when their count is high [66,67]. Testosterone binds to androgen receptors, which activate several different intracellular pathways and encourage lipolysis [66,67]. As a result, triglycerides are broken down into FFAs that are released into the bloodstream [66]. Despite the higher rate of fat being broken down, men often appear “apple” or “android”-shaped due to the VAT accumulated around their abdomen. This is because the FFAs that are circulating through the bloodstream continue down towards VAT deposits, where they are once again taken up and re-esterified (Figure 4) [45]. Additionally, as males age, their testosterone levels begin to decrease, and the rate of lipolysis in both subcutaneous and VAT decreases as well, leading to a large accumulation of fat in the center of the body. Many researchers believe that this accumulation of central fat may be one of the reasons that males typically develop T2D at a lower BMI than females in the same age range [68]. Essentially, females have a greater abundance of estrogen, which encourages the adipogenesis of subcutaneous adipose tissue while limiting the amount of FFAs taken up by VAT. Males, on the other hand, have a larger number of androgens, which encourage lipolysis, leading to FFAs flowing to the abdomen to be taken up by VAT. Hormones play a complex role in the accumulation of various adipose tissues, especially because they change as people age, but they also establish foundational differences in the location of fat deposition. 

As previously mentioned, the adipose tissue expandability hypothesis is also applicable when analyzing FFA storage capacity differences between sexes. The amount of FFAs an individual can store in their subcutaneous fat cells has been proven to vary greatly, especially with sex and the location of the tissue [69]. Determining the capacity of various subcutaneous tissues depends on two major procedures: body composition measurements and adipose tissue biopsies [69]. Body composition measurements are performed using dual-energy X-ray absorptiometry, which assesses the amount of total, abdominal, and lower body fat in each participant, and computed tomography (CT), which assists in determining an individual’s VAT [69]. Adipose tissue biopsies are retrieved from the abdomen and femoral region to measure adipocyte size and lipid activity [69]. Together, these data are used to calculate the capacity of FFA storage in terms of the FFA rate of metabolism per kilogram of body weight per minute [69]. A study used these techniques to determine the capacity for FFAs by sex and location of the tissue and found that females can store about 0.37 ± 0.15 μmol·kg^−1^·min^−1^ FFAs in their upper subcutaneous adipose tissue and 0.42 ± 0.19 μmol·kg^−1^·min^−1^ in their lower subcutaneous adipose tissue [69]. These values were significantly different from those found in males, with 0.27 ± 0.18 μmol·kg^−1^·min^−1^ in their upper subcutaneous adipose tissue and 0.22 ± 0.11 μmol·kg^−1^·min^−1^ in their lower subcutaneous adipose tissues [69]. These data support the idea that females have a significantly higher capacity to store FFAs in their subcutaneous fat, so fewer FFAs circulate and move towards their VAT. The decreased FFA storage capacity in the subcutaneous fat of males is also clearly depicted here, as it leads to more FFA uptake by VAT, thus promoting VAT accumulation. FFA storage capacity is significantly impacted by sex, leading to a direct impact on the accumulation of VAT.

### 4.3. Healthy Habits

Finally, a factor that individuals can use to influence adipose tissue growth is an individual’s diet and lifestyle. A recent study found that food labeled “ultra-processed” is particularly linked to the growth of age-related VAT over subcutaneous fat [70]. Though further experimentation is necessary, they discuss that this may be due to the fact that ultra-processed food is often calorie-dense but has poor nutritional quality [70]. Another study found a link between visceral adiposity and sugar-sweetened beverages [71]. These authors found that daily consumption of sugar-sweetened beverages was positively related to an individual’s VAT-to-subcutaneous-fat ratio and suspected that fructose may be amplifying this effect [71]. Their thoughts were supported by a previous study that concluded fructose consumption was directly related to VAT deposition, especially in men, while glucose consumption was more closely related to subcutaneous fat deposition [72]. This is because when fructose is present in the bloodstream, it is absorbed into various tissues by the GLUT-5 transporter [73]. 

Increased amounts of fructose in tissues have many effects, one of which is an increase in macrophage infiltration, which leads to low-grade inflammation [73]. The inflammation subsequently increases cortisol in the blood, which increases the flow of fatty acids out of subcutaneous adipocytes and allows for more to be taken in by visceral adipocytes [73]. Saturated fatty acids have also been proven to increase VAT and hepatic fat. One reason for this may be that they are less oxidized and play a major part in creating triglycerides close to the liver region [74]. Changes in diet are critical, but other lifestyle aspects are also significant. One study determined that individuals who smoked and consumed alcohol were found to have more VAT than their counterparts who did not [75]. Ultimately, individuals who consume fewer “ultra-processed” foods, less fructose, and less saturated fatty acids are less at risk of accumulating VAT. Additionally, those who abstain from smoking and consuming alcohol are also less likely to accumulate VAT. Diet and lifestyle are factors within a person’s control, and making changes in these areas can prevent the growth of VAT and lower one’s risk of developing insulin resistance. Understanding the difference between the types of adipose tissues and what predisposes individuals to have one or the other may help explain the prevalence of metabolic diseases in certain groups.

## 5. Beyond BMI: Measuring Body Fat Composition

BMI is a simple calculation that relies solely on weight and height measurements and often disregards the complexities of body composition when assessing metabolic health. The equation caters to large populations but is not the best indicator of health at an individual scale because of bone and muscle mass in addition to types of adipose tissue [76]. One study evaluated the accuracy of BMI compared with bioelectrical impedance, which measures the exact percentage of body fat in an individual. They found that 30% of men and 46% of women who were not BMI-obese were actually considered obese due to their levels of body fat [77]. Another similar study was conducted, comparing BMI to dual-energy absorptiometry (DEXA). This study also found that 41% of men and 32% of women were falsely categorized as healthy while having body fat compositions that indicated that they were obese [78]. Understanding the importance of adipose tissue when assessing metabolic health, researchers from both studies also tried to derive an equation from BMI to predict the percentage of body fat in an individual. Unfortunately, in both cases, the relationship yielded a significant error [77,78]. Due to the flaws in BMI and the established influence that VAT has on T2D development, it is important to utilize body composition measurements to reveal more about a person’s metabolic health. 

Research surrounding body composition measurements emerged in the 1940s with tracer dilution measurements [79]. This assessed the distribution of fluids and ions in the body and retrieved values of total body water and extracellular water, which could be used to estimate body fat and lean body mass [79,80]. Tracer dilution measurements require scientists to choose an appropriate tracer, usually deuterium or oxygen-18 [81]. They then collect a sample, such as blood or saliva, to determine a baseline to compare to the final sample [81]. The tracer is then introduced to the body and allowed to reach equilibrium before its enrichment is measured with either mass spectroscopy or infrared spectroscopy [81]. Using the measured values, researchers are able to calculate the desired measurements with high accuracy [81]. Despite being a precise tool, tracer dilution measurements are extremely time-, labor-, and resource-intensive, making them less commonly used compared with other methods in the present day [80]. Because of these limitations, a new method called in vivo neutron activation analysis (INVA) began to be developed and experimented with. INVA directly measures elements in the human body and uses these to determine components of body composition [79,82]. In order to do this, researchers expose a subject to a beam of neutrons, which interact with elements within tissues to form new chemical states [83]. These elements work to return to their stable states by releasing gamma rays [83]. The activity of these rays is measured, which gives rise to the element’s abundance [83]. Once the abundance has been assessed, it can be used to calculate proteins, minerals, and fats within the body, which ultimately allows for the calculation of body composition [83]. Unfortunately, there are many factors that can impact the accuracy of this process, such as uniform flow to subjects, detectors, dosage, and calibration [84]. Due to this, the use of imaging methods has become more popular, with techniques such as DEXA, CT scanning, and magnetic resonance imaging (MRI) at the forefront [79]. 

CT scanning is an imaging technique that involves creating cross-sectional images of the body to visualize its composition. Initially, the scanner will emit several X-ray beams that will attenuate to different extents based on the type of tissue it interacts with [85]. This degree of attenuation identifies the density of tissues, which is what leads to the development of an image [85]. In addition to the image, it can also reveal quantitative data such as the mass of muscle or a type of adipose tissue, making it a valuable tool for analyzing body composition, specifically fat distribution [85]. This method is highly precise and yields consistently accurate measurements, but it is expensive and lacks accessibility. This makes it less ideal for clinical assessments and research compared with other body composition imaging techniques. 

MRI is an imaging technique that utilizes magnetic fields and radio waves to develop images of the body’s structures. Tissues being scanned contain many hydrogen atoms, which align themselves with the magnetic field when exposed to the MRI [86]. Then, a radio wave pulse is released, which disrupts the alignment of the hydrogen atoms by making them absorb energy [86]. These atoms quickly return to their initial alignment when the radio waves are turned and release small amounts of energy [86]. The energy released is then relayed back to the MRI scanner, which uses this input to create cross-sectional images of the body [86]. MRI is also very accurate, making it extremely useful, especially in clinical settings, but there is still another means of analyzing body composition used more commonly in research due to various factors. 

Despite the nuances of CT scanning and MRI, DEXA has often been regarded as the best way to assess an individual’s body composition due to its versatility and accuracy. DEXA works by emitting radiation energies that are variably weakened based on density, anatomical structure, and intensity of the energy [87]. By doing this, it retrieves an “R-value,” which is described as the ratio between attenuation coefficients at two different energy levels [87]. This R-value will vary based on the tissue being assessed, but generally, the R-value for an individual with a high fat percentage would be lower compared with that of an individual with a higher percentage of lean mass [87]. DEXA also separates the body into three main components: bone mineral content, fat mass, and lean mass [87]. Because of this, researchers and healthcare professionals can gain a clearer understanding of an individual’s body composition. DEXA especially stands out when compared with MRI and CT scanning because it is relatively more accessible for patients, more comfortable due to the short duration of the scan, and safer due to the comparatively low dose of radiation that patients are exposed to [87]. Because of its precision, accessibility, and versatility, DEXA is an ideal method to determine the amount of VAT an individual has accumulated. It is important to emphasize that anthropometric measures such as BMI are still extremely valuable due to their ease and simplicity, but when VAT has proven to play such a large role in T2D development, it is critical to use BMI in conjunction with body composition measurements such as DEXA to curb the rise of this disease. 

## 6. Conclusions

As the number of T2D cases has soared to an all-time high, scientific comprehension of the condition has surged alongside it. T2D has previously been regarded as a metabolic condition associated with BMI obesity that results in faulty insulin sensitivity and decreased glucose uptake. Despite this, body composition, specifically VAT, has proven to be one of the largest driving factors behind T2D in both obese and non-obese individuals. It is true that obese individuals are at high risk for T2D development as they release more insulin after consuming glucose, which often leads to impaired pancreatic β -cell function and insulin resistance. However, non-obese individuals compose a substantial portion of T2D cases due to the accumulation of VAT deposits. VAT releases large concentrations of FFAs into the bloodstream, which flow to various other tissues and promote insulin resistance. This may be attributed to the tissue’s poor ability to take in and store circulating FFAs compared with other adipose tissues, such as subcutaneous or brown fat. The interplay of genetics and sex differences significantly determines the distribution of subcutaneous versus VAT, leaving much room for variation. Individuals from several countries, particularly in Asia, exhibit greater VAT despite maintaining a low BMI. Moreover, males demonstrate a biological predisposition for greater VAT accumulation due to their increased androgen production, reduced estrogen production, and diminished ability to uptake and store FFAs. This susceptibility only increases with age and a reduced ability to produce necessary hormones and store circulating FFAs. Diet and lifestyle are additional factors that may be adjusted to discourage the development of VAT. Individuals who consumed fewer processed foods, less fructose, less saturated fatty acids, less alcohol, and smoked less have shown much less VAT accumulation. Given its profound impact on insulin resistance in surrounding tissues, the current research emphasizes that VAT is a paramount indicator of T2D. Considering the role of VAT in T2D development and addressing individual body composition is integral to modern treatment as the prevalence rate of this disease continues to increase around the world. 

## Figures and Tables

**Table 1:** A list of the complications of T2D on various organ systems, including the nervous system, cardiovascular system, immune system, and urinary system.

Organ System	Physiological Effects/Complications of T2D
Nervous System	Faster progression of brain atrophy with more white matter lesions [10] Decreased gray matter, white matter, and hippocampus volume [11] Decreased/abnormal functional connectivity in the resting state [10] Significantly increased risk of stroke and stroke-related death [12] Decrease in sensory function, especially in the lower extremities (diabetic neuropathy) [13]
Cardiovascular System	Increased risk of heart failure and death from heart failure [14] Increased risk of cardiovascular disease (CVD) [15] Increased risk of hypertension [16] Increased risk of developing atrial fibrillation and thromboembolism [17]
Immune System	Dysfunction of the innate immune response (includes macrophages and neutrophils) [18] Dysfunction of the adaptive immune response (includes T cells) [18]
Urinary System	Increased urinary bladder wall thickness [19] Abnormal bladder sensation, incontinence, and overall bladder dysfunction [20] Increased risk of diabetic kidney disease and decrease in glomerular filtration rate [21] Increased risk of urinary tract infections with more severe symptoms and complications [22]
